# Antioxidant Protection against Curative and Palliative Doses of Ionizing Irradiation in Human Blood Decreases with Aging

**DOI:** 10.1100/2012/982594

**Published:** 2012-05-02

**Authors:** Jelena Kasapović, Vesna Stojiljković, Ljubica Gavrilović, Nataša Popović, Zorka Milićević

**Affiliations:** Laboratory of Molecular Biology and Endocrinology, Vinča Institute of Nuclear Sciences, University of Belgrade, Mike Petrovića Alasa 12-14, P.O. Box 522, 11 000 Belgrade, Serbia

## Abstract

Reactive oxygen species (ROS) are independently recognized to play a significant role in radiation-induced damage on healthy tissue and in aging process. However, an age-related alteration of antioxidant (AO) system in radiation response in humans is poorly investigated. The aim of this paper was to evaluate the irradiation effects on the activities and expression of AO system in the blood of healthy women during aging. Blood samples were irradiated with curative and palliative doses of 2 Gy or 9 Gy **γ**-rays. AO capacity for detoxification of O_2_•^−^ and H_2_O_2_ in response to 2 Gy **γ**-irradiation decreases in women above 58 years, while in response to 9 Gy shows signs of weakening after 45 years of age. Due to reduction of AO capacity during aging, cytotoxic effects of curative and palliative doses of irradiation, mediated by ROS, may significantly increase in older subjects, while removal of H_2_O_2_ excess could reduce them.

## 1. Introduction

Radiotherapy of cancer tumors is based on the possibility of tumor cell death induction mediated by the direct damages of DNA molecules, as well as by the high local production of reactive oxygen species (ROS) [1]. Radiochemical changes occurs within 10–13 seconds, including the generation of O_2_•^−^, •OH, and H_2_O_2_, and other free radicals attributable to chemical interactions between high-energy electrons, photons, and the molecular targets of oxygen and water within cells. The secondary radicals are formed by the interaction of •OH with organic molecules [2]. Oxidative damage of biomolecules formed by the action of ROS, which exceeds the antioxidant (AO) and DNA repair capacity and leads to a cell death, is a favorable outcome for the tumor cells, but is a highly undesired event for the healthy tissues [3]. Severe side effects commonly arising from radiotherapy often prevent patients from completing the treatment course. Thus, scavenging ROS and inhibiting lipid peroxidation are among possible key target activities for developing successful radioprotection strategies. Since the degree of radiation-induced damage on healthy tissue determines the quality of life after radiotherapy, AO enzymes involved in radioprotective mechanisms [4, 5], as well as in mechanisms of radiosensitivity and radioresistance [6–10] are the subject of intensive researches.

Our previous research showed that the activity of manganese superoxide dismutase (MnSOD) is inversely correlated with the yield of *γ*-radiation-induced micronuclei *in vitro *in healthy human blood lymphocytes, suggesting a base for a rapid predictive assay of radiosensitivity in a clinical setting [7]. Our results also showed that activity of SOD, particularly MnSOD, significantly contributes to the relative biological effectiveness and radiosensitivity to proton irradiation *in vitro *as measured by the dose-dependent production of dicentrics and micronuclei in healthy human lymphocytes [11].

ROS (oxidative stress) is independently recognized to play a significant role in radiation-induced damage on healthy tissue [12, 13] and in aging process [14, 15]. However, an age-related modulation of AO system and AO enzyme expression in radiation response in humans is poorly investigated. The only obtainable report was given by Lenton and Greenstock [16], who showed inverse relationship between human plasma radioprotective ability to 26 Gy *γ*-irradiation and donor age, based on total plasma AO capacity. Thus, in this work we investigated the AO enzyme activities of copper, zink superoxide dismutase (CuZnSOD), catalase (CAT), glutathione peroxidase (GPx), and glutathione reductase (GR), as well as concentration of glutathione (GSH) in blood cells of healthy women after *in vitroγ*-irradiation of blood. Women were divided into three age groups, younger then 45 years, aged 45–58 years, and older then 58 years. The level of CuZnSOD protein was measured to estimate whether the changes in activities were related to the protein level of this enzyme, since in our previous work we found that radiotherapy reduces CuZnSOD activity through the mechanism of reduced synthesis of this enzyme [17]. Doses of 2 Gy and 9 Gy *γ*-rays were chosen as a representative for daily clinical doses of 1.8–2 Gy in curative radiotherapy and 8–10 Gy in palliative radiotherapy. They correspond to cumulative concentrations of 2 × 10^6^ M and 9 × 10^6^ M of different ROS [18]. This study is of significant importance to cancer patients since rapidly dividing cells of the blood system are highly prone to irradiation-induced damage [19]. 

## 2. Materials and Methods

### 2.1. Reagents

All reagents were purchased from Sigma (St. Louis, MO, USA), and Merck (Darmstadt, Germany). Assays for CuZnSOD, GPx, GR, and GSH were purchased from BioxytechR Assays (OXIS International Inc., Portland, OR, USA). Rabbit anti-CuZnSOD polyclonal antibody (SOD-100) and alkaline phosphatase-conjugated goat anti-rabbit IgG (SAB-301) were purchased from Stressgen Biotechnologies (Victoria, Canada).

### 2.2. Subjects

The study included fifty-four healthy women, selected from the routine control examinations of employees and retirees, in the Department of Radiological Health Protection of Vinča Institute of Nuclear Sciences, University of Belgrade, Belgrade, Serbia. They were divided according to their age into the age groups of younger than 45 years (number of subjects, *n* = 13, median age in years: 33, range in years: 24–42), 45–58 years (*n* = 23, median age: 53, range: 45–58) and above 58 years (*n* = 18, median age: 71, range: 63–80). The subjects in the youngest age group had regular menstrual cycles, those in the middle-aged group were menopausal, while those in the oldest age group were postmenopausal. Participants were nonsmokers, and they used no alcohol consumption, hormones, oral contraceptives, or dietary supplements with antioxidants. None of the subjects had diseases such as diabetes mellitus, rheumatoid arthritis, liver disorders, or any malignancies. According to the ethical guidelines of the Helsinki Declaration, informed consent was obtained from all participants and the protocol used in this study was approved by the Ethics Committee of Vinča Institute of Nuclear Sciences, University of Belgrade, Belgrade, Serbia.

### 2.3. Irradiation

Blood samples were obtained after an overnight fast by venous arm puncture in lithium-heparinized tubes. Each blood sample was divided into the triplicate aliquots and placed into plastic syringes under the sterile conditions (Holten Laminar Air, Heto-Holten A/S, Allerod, Denmark). They were positioned in a plexiglas container 15 × 15 cm^2^, and irradiated with 2 Gy or 9 Gy dose using ^60^Co as a source of *γ*-radiation (Cobaltron- Therapeutic gamma source, Electronic Industry Niš, Serbia). Dose-rate was 0.333 Gy/min and the dimension of radiation field was 15 × 15 cm^2^. Samples were irradiated at room temperature. Nonirradiated samples served as controls.

### 2.4. Blood Samples

After irradiation blood sample triplicates were prepared for the *in vitro* culturing under sterile conditions (Holten Laminar Air). Blood cultures containing 0.5 mL of blood, 5 mL of RPMI-1640 medium with glutamax, and 10% fetal bovine serum, were kept for 48 h, at 37°C, and 5% CO_2_ concentration (Sanyo CO_2_ incubator, Sanyo Electric Co., Ltd., Gunma, Japan). Cells were spun down by the centrifugation at 230 g, for 5 min, at 4°C (Beckman JA20 centrifuge, Beckman Instruments Inc., Palo Alto, CA, USA), and medium and serum were removed. Cells were washed three times in cold 0.9% NaCl and centrifuged at 230 g, for 5 min, at 4°C.

Blood cells from the pellet were lysed in 2 volumes of ice-cold demineralized ultrapure water (MilliQ reagent grade water system, Millipore Corp., Bedford, MA, USA) and crude lysate were kept frozen at −70°C, before being used for clarified lysate preparations. The crude lysates were used for CAT and GPx assays and protein concentration measurements. Hemoglobin was removed from crude lysate by adding chloroform and ethanol (proportions of lysate : chloroform : ethanol was 1 : 1 : 0.6) and after centrifugation at 3000 g, for 10 min, at 4°C (Eppendorf centrifuge 5417, Eppendorf AG, Hamburg, Germany), the upper aqueous layer was collected and used for CuZnSOD activity assay, SDS-PAGE electrophoresis, and western blot analyses. After removing blood cell stroma from crude lysate by centrifugation at 8600 g, for 10 min, at 4°C (Eppendorf centrifuge 5417), clarified lysate was used for GR assay. After protein precipitation from crude lysate, with trichloroacetic acid contained in precipitation reagent (Oxis Bioxytech GSH-420 Assay; Oxis International, Inc., Portland, OR, USA; proportions for lysate : precipitation reagent was 1 : 3) and centrifugation at 10000 g, for 5 min, at room temperature (Eppendorf centrifuge 5417), supernatant was used for GSH assay.

### 2.5. Enzyme Assays

#### 2.5.1. Assay of SOD Activity

Determination of SOD activity was performed using Oxis Bioxytech SOD-525 Assay (Oxis International, Inc., Portland, OR, USA). The method is based on SOD-mediated increase of autoxidation of 5,6,6a,llb-tetrahydro-3,9,10-tryhydroxybenzo[c]fluorene in aqueous alkaline solution to yield a chromophore with maximum absorbance at 525 nm. The SOD activity is determined from the ratio of the autoxidation rates in the presence (*V*_*s*_) and in the absence (*V*_*c*_) of SOD. One SOD-525 activity unit is defined as the activity that doubles the autoxidation rate of the control blank (*V*_*s*_/*V*_*c*_ = 2).

#### 2.5.2. Assay of CAT Activity

CAT activity was determined by the method of Beutler [20]. The reaction is based on the rate of H_2_O_2_ degradation by catalase contained in the examined samples. The reaction was performed in an incubation mixture containing 1 M Tris-HCl, 5 mM EDTA, pH 8.0, and monitored spectrophotometrically at 230 nm. One unit of CAT activity is defined as 1 *μ*mol of H_2_O_2_ decomposed per minute under the assay conditions. 

#### 2.5.3. Assay of GPX Activity

GPx activity was assessed using the Oxis Bioxytech GPx-340 Assay (Oxis International, Inc., Portland, OR, USA), based on the principle that oxidized glutathione (GSSG) produced upon reduction of an organic peroxide by GPx is immediately recycled to its reduced form (GSH) with concomitant oxidation of NADPH to NADP+. The oxidation of NADPH was monitored spectrophotometrically as a decrease in absorbance at 340 nm. One GPx-340 unit is defined as 1 *μ*mol of NADH oxidized per minute under the assay conditions.

#### 2.5.4. Assay of GR Activity

Activity of GR was measured using the Oxis Bioxytech GR-340 Assay (Oxis International, Inc., Portland, OR, USA). The assay is based on the oxidation of NADPH to NADP+ during the reduction of oxidized glutathione (GSSG), catalyzed by a limiting concentration of glutathione reductase. The oxidation of NADPH was monitored spectrophotometrically as a decrease in absorbance at 340 nm. One GR-340 unit is defined as 1 *μ*mol of NADH oxidized per minute under the assay conditions.

#### 2.5.5. GSH Concentration

The concentration of GSH was measured by Oxis Bioxytech GSH-420 Assay (Oxis International, Inc., Portland, OR, USA), which is based on the reaction of 4-chloro-1-methyl-7-trifluoromethylquinolinium and all thiols to form thioethers. Upon addition of base to raise the pH above 13, a *β*-elimination specific to the GSH-thioether results in production of the chromophoric thione, which is measured at 420 nm.

The enzyme assays and concentration measurements were monitored spectrophotometrically (Perkin Elmer spectrophotometer, *λ*25, Perkin Elmer Instruments, Norwalk, CT, USA). The enzyme activities were expressed in U or mU per milligram of total cell protein. GSH and LP concentration was expressed in nmol/mg protein and pmol/mg protein, respectively. Determination of protein concentration was performed by the method of Lowry et al. [21] and expressed in mg/mL.

### 2.6. Electrophoresis and Western Blot

For the SDS-PAGE electrophoresis equal amounts of protein were dissolved in SDS-PAGE sample loading buffer and electrophoresed in 10% polyacrylamide gel (Mini-Protean 3 Cell, Bio-Rad Laboratories, Inc., Hercules, CA, USA), according to Laemmli [22]. For Western blot analysis, the proteins were transferred to nitrocellulose membranes (Trans- Blot SD Semi- Dry Electrophoretic Transfer Cell, Bio-Rad, Hercules, CA, USA). Non-specific binding sites on membranes were blotted with TBST (10 mM Tris, 150 mM NaCl, 0.1% Tween 20) containing 1% BSA and then probed with rabbit anti-CuZnSOD polyclonal antibody (SOD-100, Stressgen Biotechnologies, Victoria, Canada) and rabbit anti-actin antibody (C-11: 1615, Santa Cruz Biotechnology, Santa Cruz, CA, USA). Alkaline phosphatase-conjugated goat anti-rabbit IgG (SAB-301, Stressgen Biotechnologies, Victoria, Canada) was used for detection purposes. Each blot was ran in triplicate and scanned. The density of bands was determined by ImageJ processing program, normalized to the level of actin and expressed as percent of value found in the control samples, which were consider as 100%.

### 2.7. Statistical Analyses

Statistical analyses were performed by using the statistical software package OriginPro 8.0. Variations of AO parameters were tested with Student's paired *t*-test. Data were tested at a statistical significance level of *P* < 0.05 and expressed as mean  ±  SEM.

## 3. Results

### 3.1. Effects of *In Vitro* *γ*-Irradiation on AO Status in the Blood Cells of Healthy Women

**Figure 1:**
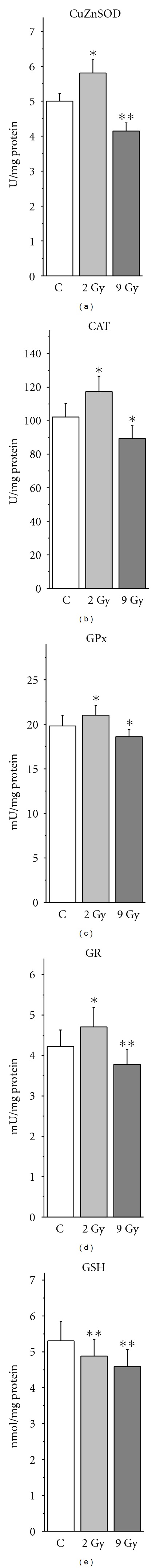
AO enzyme activities and concentration of GSH in blood cells nonirradiated (C) and irradiated with 2 Gy and 9 Gy of *γ*-radiation, in healthy women younger than 45 years. The data are given in mean values ± SEM; *P < 0.05, **P < 0.01 in comparison to C (student's paired *t*-test).

The dose of 2 Gy *γ*-irradiation, in the age group under 45 years, increased the activities of SOD (5.00 ± 0.22 versus 5.81 ± 0.38 U/mg prot., Student's paired *t*-test: *P* < 0.05), CAT (102.17 ± 7.99 versus 117.37 ± 9.11 U/mg prot., *P* < 0.05), GPx (19.82 ± 1.19 versus 21.02 ± 1.10 mU/mg prot., *P* < 0.05), and GR (4.22 ± 0.41 versus 4.71 ± 0.48 mU/mg prot., *P* < 0.05), and lowered the level of GSH (5.31 ± 0.54 versus 4.88 ± 0.47 nmol/mg prot., *P* < 0.01), when compared to nonirradiated control values (Figure 1).

**Figure 2:**
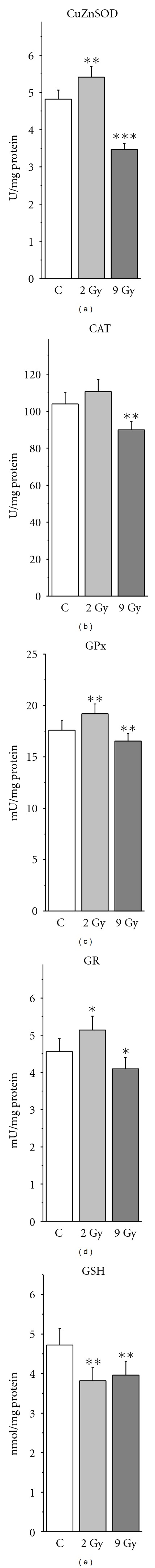
AO enzyme activities and concentration of GSH in blood cells nonirradiated (C) and irradiated with 2 Gy and 9 Gy of *γ*-radiation, in healthy women aged 45–58 years. The data are given in mean values ± SEM; *P < 0.05, **P < 0.01, ***P < 0.001 in comparison to C (student's paired *t*-test).

In the group aged 45–58 years, the dose of 2 Gy increased the activities of SOD (4.82 ± 0.24 versus 5.41 ± 0.28 U/mg prot., *P* < 0.01), GPx (17.60 ± 0.93 versus 19.21 ± 0.93 mU/mg prot., *P* < 0.01), and GR (4.56 ± 0.35 versus 5.14 ± 0.37 mU/mg prot., *P* < 0.05), had no effect on the activity of CAT (103.99 ± 6.29 versus 110.60 ± 6.66 U/mg prot., *P* > 0.05), and decreased the level of GSH (4.72 ± 0.42 versus 3.82 ± 0.33 nmol/mg prot., *P* < 0.01) in comparison to control values (Figure 2).

**Figure 3:**
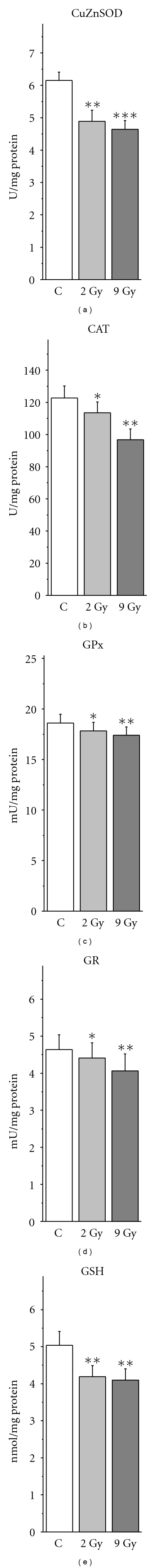
AO enzyme activities and concentration of GSH in blood cells nonirradiated (C) and irradiated with 2 Gy and 9 Gy of *γ*-radiation, in healthy women above 58 years. The data are given in mean values ± SEM; *P < 0.05, **P < 0.01, ***P < 0.001 in comparison to C (student's paired *t*-test).

In the age group above 58 years, the equal dose of *γ*-rays decreased the activities of SOD (6.15 ± 0.26 versus 4.89 ± 0.34 U/mg prot., *P* < 0.01), CAT (122.66 ± 7.51 versus 113.51 ± 6.70 U/mg prot., *P* < 0.05), GPx (18.60 ± 0.88 versus 17.84 ± 0.84 mU/mg prot., *P* < 0.05), and GR (4.64 ± 0.40 versus 4.41 ± 0.41 mU/mg prot., *P* < 0.05) and lowered the level of GSH (5.04 ± 0.37 versus 4.19 ± 0.30 nmol/mg prot., *P* < 0.01) (Figure 3).

The dose of 9 Gy *γ*-irradiation uniformly decreased the activities of AO enzymes and the level of GSH in all examined age groups of subjects, when compared to nonirradiated control values. The values of  AO parameters for the control samples versus irradiated samples were as follows:

- in the group under 45 years: SOD (5.00 ± 0.22 versus 4.15 ± 0.24 U/mg prot., Student's paired *t*-test: *P* < 0.01), CAT (102.17 ± 7.99 versus 89.41 ± 7.66 U/mg prot., *P* < 0.05), GPx (19.82 ± 1.19 versus 18.16 ± 0.79 mU/mg prot., *P* < 0.05), GR (4.22 ± 0.41 versus 3.78 ± 0.37 mU/mg prot., *P* < 0.01), and GSH (5.31 ± 0.54 versus 4.59 ± 0.47 nmol/mg prot., *P* < 0.01) (Figure 1);
- in the group aged 45–58 years: SOD (4.82 ± 0.24 versus 3.47 ±  0.16 U/mg prot., *P* < 0.001), CAT (103.99 ± 6.29 versus 89.95 ± 4.70 U/mg prot., *P* < 0.01), GPx (17.60 ± 0.93 versus 16.53 ± 0.73 mU/mg prot., *P* < 0.01), GR (4.56 ± 0.35 versus 4.10 ± 0.30 mU/mg prot., *P* = 0.05), and GSH (4.72 ± 0.42 versus 3.96 ± 0.35 nmol/mg prot., *P* < 0.01) (Figure 2);
- in the group above 58 years: SOD (6.15 ± 0.26 versus 4.64 ± 0.27 U/mg prot., *P* < 0.001), CAT (122.66 ± 7.51 versus 96.71 ± 6.76 U/mg prot., *P* < 0.01), GPx (18.60 ± 0.88 versus 17.41 ± 0.81 mU/mg prot., *P* < 0.01), GR (4.64 ± 0.40 versus 4.06 ± 0.46 mU/mg prot., *P* < 0.01), and GSH (5.04 ± 0.37 versus 4.10 ± 0.30 nmol/mg prot., *P* = 0.01) (Figure 3).

### 3.2. Effects of *In Vitro* *γ*-Irradiation on SOD Protein Level in the Blood Cells of Healthy Women

**Figure 4:**
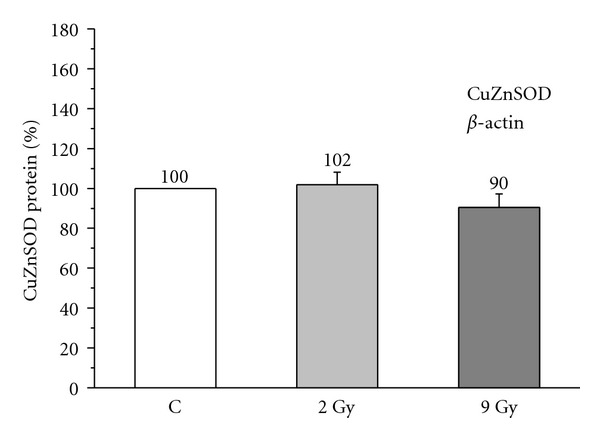
Relative level of CuZnSOD protein in blood cells nonirradiated (C) and irradiated with 2 Gy and 9 Gy of *γ*-radiation, in healthy women younger than 45 years. Relative values are expressed as percent of value found in the nonirradiated control group, which is considered as 100% (student's paired *t*-test).

**Figure 5:**
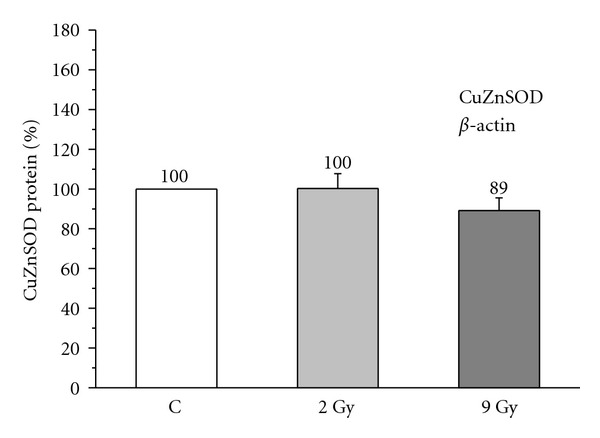
Relative level of CuZnSOD protein in blood cells nonirradiated (C) and irradiated with 2 Gy and 9 Gy of *γ*-radiation, in healthy women aged 45–58 years. Relative values are expressed as percent of value found in the nonirradiated control group, which is considered as 100% (student's paired *t*-test).

**Figure 6:**
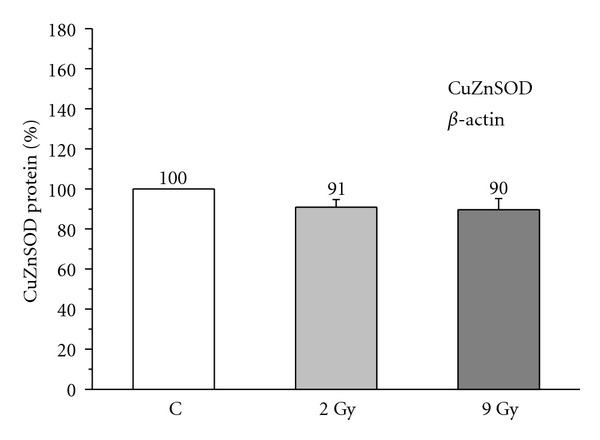
Relative level of CuZnSOD protein in blood cells nonirradiated (C) and irradiated with 2 Gy and 9 Gy of *γ*-radiation, in healthy women above 58 years. Relative values are expressed as percent of value found in the nonirradiated control group, which is considered as 100% (student's paired *t*-test).

A dose of 2 Gy, 48 hours after *γ*-irradiation, did not significantly change the level of blood cells SOD protein, in the group of subjects younger than 45 years (101.83 ± 6.32%, Student's paired *t*-test: *P* > 0.05; Figure 4), neither in the group aged 45 to 58 years (100.22 ± 7.57%, *P* > 0.05; Figure 5), nor in the group older than 58 years (90.90 ± 3.76%, *P* > 0.05; Figure 6), when compared to the age-matched nonirradiated controls (100%).

Dose of 9 Gy, 48 hours after *γ*-irradiation, also, did not significantly affect protein levels of this enzyme in the blood cells of the youngest (90.43 ± 6.79%, *P* > 0.05; Figure 4), middle-aged (89.12 ± 6.41%, *P* > 0.05; Figure 5), or elderly subjects (89.63 ± 5.61%, *P* > 0.05; Figure 6) in comparison with the age-matched nonirradiated controls (100%).

## 4. Discussion

Results of this work showed that AO response to 2 Gy of *γ*-radiation in blood cells of healthy women was similar in age groups younger than 45 years and aged 45–58 years. It involved increased activities of CuZnSOD, CAT, GPx, and GR and lowered level of GSH (Figures 1 and 2). Contrary, in the age group above 58 years the same dose of *γ*-rays lowered the activities of SOD, CAT, GPx, and GR, as well as the level of GSH (Figure 3). A dose of 9 Gy uniformly suppressed the activities of AO enzymes and lowered the level of GSH in healthy women independently of age. Suppressive effects of this dose on CuZnSOD, CAT, and GPx activity were more pronounced in groups aged 45–58 years and above 58 years (Figures 2 and 3), when compared to age group younger then 45 years (Figure 1). This shows that AO protective capacity in response to acute *γ*-radiation depends on age in women, and in a case of 2 Gy dose significantly decrease after 58 years of age, while in a case of 9 Gy dose shows signs of slight weakening already after 45 years of age.

Under oxidative stress conditions, increased level of ROS can damage the variety of biomolecules, including AO enzymes. Increased rate of ROS production commonly elicits, as a response, an increase in activities of AO enzymes. Still, under high rate of ROS input, the enzyme inactivation prevails, leading to reduced AO enzyme activities [23, 24] and to autocatalysis of oxidative damage process. Thus, cumulative increase of ROS induced by aging process and acute *γ*-irradiation may be the cause for observed decrease of AO protective capacity in response to both doses of *γ*-irradiation.

Free radicals and peroxides can be directly removed by the action of reduced GSH, which is the main endogenous soluble antioxidant and the key regulator of intracellular redox status in mammalian cells [25]. GSH can act through formation of a disulfide mixture or by oxidation to GSSG, thus preventing damaging effects on tissues caused by hydrogen and organic peroxides [26]. A decreased level of GSH may cause increased oxidative damage, both through impairment of its direct antioxidant actions and through indirect action, *via *decreasing GPx activity, where it is necessary as a cosubstrate [27]. Protective role of GPx against radiation stems from observation that GPx activity correlates with radioresistence in several human cell lines [9]. It appears that the reduction of GSH level in response to a dose of 2 Gy in the youngest and middle-age group of women is the consequence of its increased consumption, judged by increased activity of GPx (Figures 1 and 2). Reduction of GSH level in response to a dose of 2 Gy in the oldest group (Figure 3), as well as in response to a dose of 9 Gy in all age groups of women is probably due to its decreased regeneration by GR (Figures 1, 2, and 3). This confirms our previous results [28, 29], showing important role of the glutathione redox cycle in AO protection against oxidative stress in ionizing-radiation exposure and aging process.

ROS (oxidative stress) are recognized to play a significant role in functional degeneration of somatic cells during aging process [15]. Concerning the alteration of AO enzymes during aging process, our previous work showed age-related decrease of CuZnSOD and CAT activities in blood cells of healthy women aged 45–58 years and above 58 years when compared to women younger then 45 years [28]. Other reports also showed age-related decrease of SOD activity in erythrocytes [30–32] and whole blood [33] and decrease of CAT activity in blood [34] in healthy humans. Age-dependent diminishment in AO enzyme activity may be due to a progressive enzyme inactivation by its product [23]; for example, the production of mitochondrial H_2_O_2_ increases with aging [35]. Also, it may be due to an increase in the glycation of enzyme [36], deficiency of ions important for maintaining the enzyme structure and catalytic function [37], and disturbance of glutathione redox system [38]. These data support our present results, indicating that age-related decrease in AO capacity may affect AO response to acute *γ*-irradiation.

Although ROS (oxidative stress) are independently recognized to play a significant role in radiation-induced damage of healthy tissue [39] and in aging process [14], age-related modulation of AO system in radiation response in humans was poorly investigated. Investigation on humans aged 30–80 years reported inverse relationship between plasma radioprotective ability to *in vitro* 26 Gy *γ*-irradiation and donor age, based on total plasma AO capacity [16]. Study on gerbils observed that AO potential of tissue homogenates from older animals had a reduced ability to protect against radiation-induced oxidative damage provoked by 60 Gy X-irradiation, measured by protein oxidation susceptibility. The same study showed that activities of SOD, CAT, GPx, and concentration of GSH did not exhibit a uniform pattern of age-related changes [40]. Our previous report indicated that radiotherapy with a fraction size of 2 Gy X-radiation for breast tumors and regional lymph nodes, includes age-related decrease in AO capacity for elimination of H_2_O_2_ (decrease of GPx, GR activities, and GSH level)_._ This caused the occurrence of oxidative damages in blood cells of patient above 58 years, when compared to patients aged 45–58 years [29]. Our previous and present results indicate that age-related decrease in AO capacity affects not only the AO response to acute *γ*-irradiation with 2 Gy, but also to repeatedly delivered dozes of 2 Gy X-irradiation during radiotherapy. This is of particular interest for ionizing radiotherapy, because about two-thirds of X-ray and *γ*-ray damage is caused by their indirect action, *via* ROS, that kill tumor cells but threaten the integrity and survival of surrounding normal cells [39, 41].

The alteration of AO enzyme expression in aging process and radiation response in humans is also poorly documented. *In vitroγ*-irradiation of blood of breast cancer patients with doses of 2 Gy and 9 Gy showed increased expression of MnSOD and CAT, and unchanged expression of CuZnSOD in leukocytes [42]. Higher activities and expression of glutathione-S-transferase (GST) and CAT were recorded in radioresistant variant of human glioblastoma cell line after *in vitro* fractionated *γ*-irradiation with 3 Gy [10]. In our previous work we observed that the age-related decrease in CuZnSOD activity in blood cells of healthy humans is most likely caused by the posttranslational chemical modification, without changes in protein synthesis of this enzyme [28]. We also found that breast cancer radiotherapy with a fraction size of 2 Gy X-radiation provoked elevation of blood cells CuZnSOD activity in patients aged 45–58 years and above 58 years, caused by the increase in protein synthesis of this enzyme [17]. Results in this work show that modulation of CuZnSOD activity in response to 2 Gy and 9 Gy doses of acute *γ*-irradiation were not caused by protein level of this enzyme, which was found to be similar in aging groups (Figures 4, 5, 6). Most probably it is due to post-translational chemical modification of this enzyme under the changed redox environment provoked by ionizing radiation. Although statistically insignificant, the increase in CuZnSOD activity coincided with its unchanged protein levels, while decrease in activity coincided with its decreased protein levels for about 10%. It may be noted that, while an acute 2 Gy ionizing irradiation modifies CuZnSOD activity in blood cells through faster mechanism of post-translational chemical modification of enzyme, repetitively delivered 2 Gy ionizing irradiation during radiotherapy [17] modifies CuZnSOD activity through slower, but more efficient mechanism of synthesis of this enzyme.

In conclusion, our results seem to be the first report of deleterious contribution of aging process to decrease of blood cell AO enzyme activities and GSH concentration in ionizing-radiation response in humans. The protective AO capacity for detoxification of O_2_•^−^ and H_2_O_2_ in response to 2 Gy of *in vitroγ*-irradiation decreases in women above 58 years, due to decrease in CuZnSOD, CAT, GPx, and GR activities. Also, AO capacity for detoxification of O_2_•^−^ and H_2_O_2_ in response to 9 Gy dose shows signs of weakening after 45 years of age, due to more pronounced decrease in CuZnSOD, CAT, and GPx activities. Thus, AO response to both doses seems to be age dependent. Modulation of CuZnSOD activity in response to both doses of acute *γ*-irradiation was not caused by protein level of this enzyme and most probably is due to post-translational chemical modification of this enzyme. This suggests that, as a result of aging process, decreased AO protection which maintains redox homeostasis [43, 44], may significantly affect the healthy cell response to curative and palliative doses of therapeutic irradiation, through toxic and/or redox regulative effects of ROS. Also, application of antioxidants that remove the excess of H_2_O_2_ possibly could reduce these side effects.
